# Retrotransposon-Derived Double-Stranded RNA as a Driver of Reactive Astrogliosis in Tauopathy

**DOI:** 10.1093/geroni/igaf122.4003

**Published:** 2025-12-31

**Authors:** Alyssa Cavalier, Paulino Ramirez, Bess Frost

**Affiliations:** Brown University, Providence, Rhode Island, United States; Brown University, Providence, Rhode Island, United States; Brown University, Providence, Rhode Island, United States

## Abstract

Nearly half of the human genome is composed of retrotransposons, virus-like sequences thought to be genetic fossils from ancient viral infections. Due to their likely viral origins, retrotransposons are typically highly suppressed; however, retrotransposons become activated in neurodegenerative diseases including Alzheimer’s disease and other tauopathies. Pathological forms of tau cause heterochromatin decondensation, leading to increased transcription of previously silenced retrotransposons. While much of the work on retrotransposons focuses on their potential to propagate throughout the genome, retrotransposon-derived products such as double-stranded RNA (dsRNA) can drive toxicity through innate immune activation. Prior work in our lab found elevated levels of astrocytic dsRNA and MDA5 (a dsRNA sensor) in human tauopathy and tau transgenic mice; additionally, pan-neuronal overexpression of Dicer-2 reduced total dsRNA burden in tau transgenic Drosophila. These data suggest neuronal origin of dsRNA that accumulates in astrocytes in tauopathy. Using iPSC-derived neurons carrying the IVS10 + 16 MAPT mutation, we observed increased retrotransposon transcripts via RNA-seq when compared to isogenic controls; however, we do not know if neurons expressing pathogenic tau have elevated dsRNA levels in vitro. RNA-seq analyses also show a broad dysregulation of pathways related to innate immune activity. Our preliminary data suggest that dsRNA burden is elevated in tau mutant iPSC-derived neurons, as well as increased expression of antiviral dsRNA sensors such as TLR-3. Current analyses are focused on downstream immune signaling pathways and potential interaction with dsRNA in tau mutant neurons. Future research aims to characterize potential modes of neuronal dsRNA release and detection by cultured astrocytes.

